# Primary amenorrhea after bone marrow transplantation and adjuvant chemotherapy misdiagnosed as disorder of sex development: A case report

**DOI:** 10.1097/MD.0000000000005190

**Published:** 2016-11-04

**Authors:** He Huang, Qinjie Tian

**Affiliations:** Department of Obstetrics and Gynecology, Peking Union Medical College Hospital, Chinese Academy of Medical Sciences, Beijing, China.

**Keywords:** bone marrow transplantation, chemotherapy, disorders of sex development, primary amenorrhea, primary ovarian insufficiency

## Abstract

**Introduction:**

Disorders of sex development (DSD) is a congenital condition in which the development of chromosomal, gonadal or genital sex is atypical. Majority of patients present clinical characteristics of primary amenorrhea, absent secondary sex characters, and abnormal hormone level. A female appearance patient with primary amenorrhea and 46 XY karyotype seems to be solid evidences to diagnose Y-chromosome-related DSD diseases, while it is not necessarily the accurate diagnosis. We report the case of an 18-year-old girl with primary amenorrhea and 46 XY karyotype misdiagnosed as Y-chromosome-related DSD.

**Clinical findings:**

The patient has normal female reproductive organs and a disrupted pubertal development after the treatment for acute myeloid leukemia (AML). We consider that her gonads were probably functional and later impaired after AML. The clinical manifestations were not consistent with DSD. With doubts, we found that she received bone marrow transplantation (BMT) from her brother and adjuvant chemotherapy 6 years ago. Her karyotype changed from normal female to a karyotype of donor (her brother) origin after BMT.

Adjuvant chemotherapy for AML may impair her ovarian function and finally bring about disrupted puberty or primary ovarian insufficiency (POI).

**Interventions:**

We provided close follow-up.

**Outcomes:**

During the second visit, the patient had her menarche lasting 4 days without any medication.

**Conclusion:**

The present case serves as a reminder that a correct diagnosis depends on the comprehensive collection of present and past medical history, complete physical examination, and careful evaluation of related adjuvant tests. Do not presumptively judge a test and mislead reasoning. In addition, ovarian function protection should be considered for young girls having chemotherapy.

## 1 Introduction

Disorders of sex development (DSD), defined as a congenital condition in which the development of chromosomal, gonadal or genital sex is atypical, includes a wide spectrum of phenotypes.^[1]^ Majority of patients present clinical characteristics of primary amenorrhea, absent secondary sex characters, and abnormal hormone level. Some of them have unique appearance and abnormal height. A female patient complaining primary amenorrhea and 46 XY karyotype can be easily diagnosed as Y chromosome-related DSD by clinical physicians. However, it is not necessarily the right diagnosis. We here report a case of an 18-year-old girl with primary amenorrhea and 46 XY karyotype after bone marrow transplantation (BMT) and adjuvant chemotherapy misdiagnosed as DSD.

## 2 Case report

An 18-year-old girl suspected DSD was transferred to our hospital for primary amenorrhea and 46 XY chromosome karyotype. The patient presented 172 cm height, 62 kg weight, and female appearance. She had normal female genitalia with visible vaginal orifice, urethral orifice, and a normal size clitoris, but scanty pubic and axillary hairs (Tanner stage II). The breast developed automatically since the age of 12 and physical examination showed developed breasts of Tanner stage III. No family member had similar symptoms. Her medical history included bone marrow transplantation (BMT) and chemotherapy because of acute myeloid leukemia (AML) when she was 12 years old. So far there is no sign of AML recurrence.

Laboratory examination showed a 46 XY karyotype, elevated follicle stimulating hormone (FSH 18.0 mIU/mL, reference range < 10 mIU/mL), low estrogen (E_2_ 27.53 pg/mL, reference range 27.0–122.0 pg/mL), and normal luteinizing hormone (LH 6.9 mIU/mL, reference range 2.12–10.89 mIU/mL), prolactin (PRL 15.35 ng/mL, reference range < 30 ng/mL), testosterone (T 0.13 ng/mL, reference range 0.10–0.75 ng/mL), progesterone (P 0.25 ng/mL, reference range 0.38–29.26 ng/mL), and dehydroepiandrosterone sulfate (DHEA-S 3.96 μmol/L, reference range 1.77–9.99 μmol/L). The levels of adrenocorticotrophic hormone (ACTH), cortisol, renin activity (PRA), aldosterone (ALD), 17-alpha-hydroxyprogesterone (17-α-OHP), serum potassium level, and blood pressure were all in normal range. Perineal and pelvic ultrasound scans showed underdeveloped uterus and vagina, no ovary found in the pelvis, and no testis detected in the pelvis, inguinal and perineal region. Bone age was not evaluated in the patient.

Female appearance individuals with primary amenorrhea and 46 XY karyotype would be considered DSD. Typical DSD diseases include 46 XY, pure gonadal dysgenesis (46XY PGD), androgen insensitivity syndrome (AIS) and 46 XY 17 alpha-hydroxylase/17, 20-lyase deficiency (17OHD). 46XY PGD patients presented obvious elevated FSH (usually >40 mIU/mL) with low E_2_ and T; they had small uterus, streak gonads, and infantile female genitalia, while no automatic breast development and pubic hair. Complete AIS patients presented normal female genitalia and automatic breast development with normal FSH level and significantly elevated T and slightly lower E2 compared with normal adult woman. They had well-developed testes either located in pelvis, inguinal canal, or labium major. The 46XY 17OHD patients presented obvious elevated FSH and P, low T, and E_2_. Complete 17OHD patients usually had primary amenorrhea without female second sex characteristics, hypertension, and hypokalemia as a result of lower synthesis of T and E2, elevated ACTH, and mineral corticosterone; they had underdeveloped gonad, female genitalia, and no uterus. However, in this patient, she already presented some female pubertal characteristics. Her mildly elevated FSH, low E_2_, P and T, normal ACTH, PRA, ALD and 17-α-OHP, normal blood pressure and serum potassium level, underdeveloped uterus and no testes in the pelvis, inguinal and perineal region were not identical with Y-chromosome-related DSD diseases.

With the doubts, we noticed her BMT medical history and check the whole medical record carefully. In 2009, she was diagnosed AML-M5 and received the HLA-matched bone marrow hematopoietic stem cell from her brother. Before BMT, she had bone marrow aspiration (BMA) and bone marrow chromosome karyotype was 46, XX, *t*(2; 12)(*p*23; *q*12), *t*(11; 22)(*q*23; *q*11) (normal female karyotype with translocation of chromosome 2 and 12 and of 11 and 22, usually no significant negative effect on growth and development of carriers). However, 37 days after BMT, her bone marrow presented that 96.57% of cells were donor derived and peripheral blood test showed 95.87% donor derived. The bone marrow chromosome karyotype was changed to 46 XY (normal male karyotype). After that, she had infusion of donor NK cells for 3 times. Also, 7 months after BMT, her bone marrow presented 100% donor derived. Additionally, as adjuvant therapy for AML, she also received DAE (Ida+AraC+VP-16), improved Bu/Cy (AraC+busulfan+CTX+VM-26+MeCCNU), MTX+MD-AraC, CAG (AraC+ACR+G-CSF), and HAG (AraC+HHT+G-CSF) intravenous chemotherapy. The chemotherapy contained busulfan and cyclophosphamide (CTX), which had been proved highly toxic to ovary and probably caused primary ovarian insufficiency (POI) for her.

The changed karyotype after BMT well explained why the patient's clinical features were not consistent with the typical 46 XY karyotype DSD. The patient was normal female with normal chromosome karyotype before BMT and a primary amenorrhea. The reason of primary amenorrhea could be ovarian hypofunction associated with BMT and chemotherapy.

During the second visit, the patient had her menarche lasting 4 days without any medication. However, she had no period in the next 2 months. The rechecked sex hormone levels presented a significant change (LH: 30.3 mIU/mL, FSH: 73.25 mIU/mL, E2: 23.15 pg/mL). The markedly elevated FSH and lower E_2_ suggested poor ovarian function. We will keep following up and probably prescribed hormone replacement therapy (HRT) for her.

The case report was approved by the Institutional Review Board of Peking Union Medical College Hospital. Informed consent was given by the patient.

## 3 Discussion

DSD is a series of rare diseases including all congenital conditions in which development of chromosomal, gonadal, or genital sex is atypical.^[1]^ Majority of patients present clinical characteristics of primary amenorrhea, absent secondary sex characteristics, and abnormal hormone level. Female appearance patients with characteristics of primary amenorrhea and 46 XY karyotype seems to be solid evidences to diagnose Y-chromosome-related DSD diseases, while it is not necessarily that case. She had underdeveloped uterus, automatically developed breasts (Tanner stage III), and normal female genitalia (Tanner stage II). Blood tests showed mildly elevated FSH (18.0 mIU/mL) and low E_2_. She already presented some female pubertal characteristics automatically, which were disrupted after the treatment for AML. It means that her gonads were probably functional to produce certain amount of estrogen for pubertal development. After comprehensive collection of detailed medical history, we found that she received BMT from her HLA-identical brother 6 years ago. Her karyotype showed normal female before BMT and then changed to a karyotype of donor (her brother) origin. Our patient had her menarche without medication. Therefore, our case was a female girl with delayed puberty, not a DSD patient. In this patient, only the karyotype of hemocyte is 46 XY, but other somatic cells and germ cells are 46 XX as somatic and germ cells originated during the embryonic stage. A correct diagnosis of patient depends on the comprehensive collection of present and past medical history, complete physical examination, and careful evaluation of related adjuvant tests. Do not presumptively judge a test and mislead reasoning.

We found a study of 28 patients after sex-mismatched BMT ^[2]^ and several case reports of chromosome changes after allogeneic BMT, particularly 1 case of female chromosome changing to male karyotype.^[3]^ However, the study and case reports all focused on treatment and remission of primary diseases other than pubertal development and reproductive patterns. To our knowledge, there is no research studies on the reproductive pattern of patients after opposite sex BMT and there is no evidence currently that opposite sex BMT alone also affect the gonadal function and pubertal development. Main threats for adolescent patients involves total body irradiation and high-dose chemotherapy as adjuvant therapy for BMT, which may lead to high organ toxicity and multiple late effects.^[4–6]^ One of the serious late effects includes ovary reserve damage, which may present delayed puberty with reported incidence of 54% to 86% of female patients.^[7]^ Maturing ovarian follicles comprise a prime target for chemotherapeutic agents. Gonadal function and future fertility potential are determined by the primordial follicle reserve. Possible mechanisms of chemotherapy inducing ovarian damage include primordial follicle apoptosis and blood vessel damage.^[8]^ Evidence showed that some chemotherapy regimens had more gonadotoxic effects on ovaries than others. Alkylating agents, such as busulfan and cyclophosphamide, were highly toxic to ovary.^[9]^ The chemotherapy for this patients included busulfan and cyclophosphamide, which can act on oocytes directly or induce oocyte death indirectly via damage to somatic cells.^[9]^ Alkylating agents might have a negative impact on her ovarian function and reserve, and finally bring about delayed puberty or POI.

POI is the depletion or dysfunction of ovarian follicles with cessation of menses before the age of 40 years.^[10]^ Gonadal dysgenesis with or without the Turner syndrome could be a cause of POI, but the karyotypes of those patients are Y-chromosome absent. If FSH is elevated into the menopausal range (>30–40 mIU/mL), a diagnosis of primary ovarian insufficiency can be established. In addition, estradiol levels of <50 pg/mL indicate hypoestrogenism.^[10]^ The immediate loss of ovarian function after chemotherapy could be transient. However, the pubertal characteristics and hormone level of our patient indicate persistent hypoestrogenism. Although she had her menarche lasting 4 days without medication, but repeated hormone level tests after her menarche showed a markedly elevated FSH (73.25 mIU/mL). We would consider her ovary damage or POI. Anti-Müllerian hormone (AMH), also known as Müllerian-inhibiting substance (MIS), is secreted by the granulosa cells of ovarian follicles and a useful marker of female reproductive function. We did not evaluate her AMH level because she already presented symptoms of POI and her ovarian hypofunction could be diagnosed by significantly elevated FSH and low E_2_. The measurement of AMH is of little help in the diagnosis and treatment process. There is only a 5% to 10% chance of spontaneous pregnancy and the prognosis of POI is considered poor. The advice for her would be HRT in the future.^[4]^ The objective of HRT extends from simply symptom relief to pubertal development promotion, prevention of osteoporosis, cardiovascular diseases, and sexual health maintenance.^[10]^ We consider oral estradiol in doses of 1 to 2 mg daily to mimic a physiologic dose range and cyclic progesterone for 10 to 14 days each month to protect endometrium from endometrial hyperplasia and endometrial cancer.

As for the protection of gonad function from chemotherapy, it has been observed that prepubertal girls’ ovary function would be less affected by chemotherapy than pubertal or adult female. For pubertal and adult patients, Gonadotropin-releasing hormone (GnRH) agonists return the hypothalamic–pituitary–gonadal axis and ovary to a comparatively quiescent state, in which ovary would be less sensitive to toxic effects of chemotherapy.^[11]^ For highly gonadotoxic chemotherapeutic regimen, Kilic's study result showed that GnRH agonists could protect gonads from cyclophosphamide-induced ovarian damage by affecting germ cell apoptosis and DNA damage.^[12]^ Other recommendations for ovary function and fertility preservation include ovarian tissue banking, embryo cryopreservation, and oocyte cryopreservation. Ovary tissue banking is now being investigated and probably would be an option in the future.^[13]^

## 4 Conclusions

It is important that clinical physicians should have a complete understanding of not only patients’ clinical manifestation, but also the medical history, to help determine diagnosis and treatment. Do not presumptively judge a test and mislead reasoning. For our patient, BMT and adjuvant chemotherapy should be responsible for her blood 46 XY karyotype, delayed puberty, and amenorrhea. Protection of ovarian function and HRT should be considered for young girls having chemotherapy.
